# In vivo PET of synaptic density as potential diagnostic marker for cognitive disorders: prospective comparison with current imaging markers for neuronal dysfunction and relation to symptomatology - study protocol

**DOI:** 10.1186/s12880-024-01224-5

**Published:** 2024-02-12

**Authors:** Greet Vanderlinden, Charles Carron, Rik Vandenberghe, Mathieu Vandenbulcke, Koen Van Laere

**Affiliations:** 1 Nuclear Medicine and Molecular Imaging, Imaging and Pathology, KU Leuven, Leuven, Belgium; 2 Division of Nuclear Medicine, University Hospitals UZ Leuven, Leuven, Belgium; 3 Department of Neurology, University Hospitals UZ Leuven, Leuven, Belgium; 4 Laboratory for Cognitive Neurology, Department of Neurosciences, KU Leuven, Leuven, Belgium; 5 Research Group Psychiatry, KU Leuven, Leuven, Belgium; 6 Department of Old-Age Psychiatry, University Hospitals UZ Leuven, Leuven, Belgium; 7 Leuven Brain Institute, Leuven, Belgium

**Keywords:** Synaptic density, ^18^F-SynVesT-1, Glucose metabolism, ^18^F-FDG, PET-MR, Cognitive impairment, Dementia, Diagnosis

## Abstract

**Background:**

^18^F-FDG brain PET is clinically used for differential diagnosis in cognitive dysfunction of unclear etiology and for exclusion of a neurodegenerative cause in patients with cognitive impairment in late-life psychiatric disorders. ^18^F-FDG PET measures regional glucose metabolism, which represents a combination of neuronal/synaptic activity but also astrocytic activity and neuroinflammation. Recently, imaging of synaptic vesicle protein 2 A (SV2A) has become available and was shown to be a proxy of synaptic density. This prospective study will investigate the use of ^18^F-SynVesT-1 for imaging SV2A and its discriminative power for differential diagnosis in cognitive disorders in a head-to-head comparison to ^18^F-FDG PET. In addition, simultaneous PET/MR allows an evaluation of contributing factors and the additional value of advanced MRI imaging to FDG/SV2A PET imaging will be investigated. In this work, the study design and protocol are depicted.

**Methods:**

In this prospective, multimodal imaging study, 110 patients with uncertain diagnosis of cognitive impairment who are referred for ^18^F-FDG PET brain imaging in their diagnostic work-up in a tertiary memory clinic will be recruited. In addition, 40 healthy volunteers (HV) between 18 and 85 years (M/F) will be included. All study participants will undergo simultaneous ^18^F-SynVesT-1 PET/MR and an extensive neuropsychological evaluation. Amyloid status will be measured by PET using ^18^FNAV4694, in HV above 50 years of age. Structural T1-weighted and T2-weighted fluid-attenuated inversion recovery MR images, triple-tagging arterial spin labeling (ASL) and resting-state functional MRI (rs-fMRI) will be obtained. The study has been registered on ClinicalTrials.gov (NCT05384353) and is approved by the local Research Ethics Committee.

**Discussion:**

The main endpoint of the study will be the comparison of the diagnostic accuracy between ^18^F-SynVesT-1 and ^18^F-FDG PET in cognitive disorders with uncertain etiology and in exclusion of a neurodegenerative cause in patients with cognitive impairment in late-life psychiatric disorders. The strength of the relationship between cognition and imaging data will be assessed, as well as the potential incremental diagnostic value of including MR volumetry, ASL perfusion and rs-fMRI.

## Background

Several neurodegenerative disorders can lead to dementia, including Alzheimer’s disease (AD), frontotemporal dementia (FTD), dementia with Lewy bodies (DLB), vascular dementia and other less frequent disorders. To aid in the differential diagnosis of patients presenting with a cognitive disorder, the consequences of neuronal dysfunction and death can be measured by imaging.

In clinical practice, most often ^18^F-fluorodeoxyglucose (FDG) PET or structural magnetic resonance imaging (sMRI) (e.g. by hippocampal volume assessment in AD) are used. ^18^F-FDG PET is healthcare-reimbursed in several countries for differential diagnosis in cognitive dysfunction of unclear etiology, and shows high sensitivity and specificity [1]. However, ^18^F-FDG PET has a disadvantage that regional glucose metabolism is a composite signal, consisting of a combination of neuronal and astrocytic activity [2], is partly determined by functional interconnectivity with other brain areas, and can be increased by local neuroinflammation. As the latter is present in all forms of neurodegeneration, a specific measure of regional neuronal impairment may thus be masked. Furthermore, acquisition circumstances (e.g. glucose blood concentration [3, 4]) and sensory stimulation during the tracer uptake period (e.g. light or noise), may influence the regional activity in sensory brain areas. In particular, medial temporal hypometabolism is variable and can be absent in many patients [5–7]. ^18^F-FDG is also not specific in the differential diagnosis between psychiatric disorders and neurodegeneration such as for behavioral variants of FTD [8]. Nonetheless, some studies have shown that ^18^F-FDG PET correlates with dementia severity [9, 10], and a correlation of neurocognitive parameters with regional changes in ^18^F-FDG uptake has been demonstrated [11]. Interestingly, in patients with subjective cognitive impairment (showing no significant deficits on neuropsychological testing), heterogeneous patterns of hypometabolism can be observed [12, 13] and also in cognitively normal patients with depressive symptoms AD-like hypometabolism has been reported, independently of amyloid burden [14]. Longitudinal studies will have to further clarify the meaning of these findings. sMRI is a widely available but a less sensitive marker with also relatively low specificity [15, 16] and quantification of hippocampal volume is not widely performed in clinical routine [17, 18]. While it is known that brain perfusion is a less sensitive marker for neuronal activity changes in neurodegeneration compared to ^18^F-FDG PET, and glucose uptake impairment is an earlier biomarker [19], brain perfusion techniques such as arterial spin labeling (ASL) are investigated as absolute regional blood flow and can be easily obtained in conjunction with other primary structural MR sequences [20–22]. The reported sensitivity and specificity of ASL for discrimination of patients from healthy volunteers (HV) varies from 53 to 86% and 84–92%, respectively [23, 24]. Differences in ASL technique and small sample sizes may be the main factors for this wide range, especially in sensitivity. Nevertheless, it has been suggested that ASL could replace the need for ^18^F-FDG and allow for another PET biomarker to be measured at the same time in PET/MR [21].

Until recently, measuring synaptic density in humans required brain tissue from autopsy or surgical resection specimens. In vivo PET imaging of synaptic density has now become possible through development of radioligands with high affinity and selectivity for synaptic vesicle protein 2 A (SV2A) [25], which is the only of three isoforms that is ubiquitously and homogeneously located/expressed in synapses across the brain. ^11^C-UCB-J has been thoroughly validated and is regarded as best-in-class ligand for SV2A/synaptic density PET imaging. ^11^C-UCB-J shows good pharmacokinetics, quantification of ^11^C-UCB-J distribution volume is possible and is an vivo proxy of synaptic density [26, 27]. ^11^C-UCB-J has been used in clinical epilepsy drug trials [28]. Simplified quantification with a white matter reference is possible for easier use in patient populations [29, 30]. Furthermore, stability of synaptic density in healthy aging has been shown [31, 32]. A direct comparison in young HV, has shown differences between resting ^18^F-FDG uptake patterns and the regional distribution of synaptic density, with relative differences up to 30% with higher ^11^C-UCB-J signal especially in the hippocampus, lateral temporal cortex and cingulate, areas of particular importance in cognitive impairment [33].

Pilot studies in patients with AD show a medial temporal decrease in synaptic density of up to 40% compared to HV [34–37], while in the neocortex more moderate changes of about 10% are present whereas for ^18^F-FDG these are about 20% [38]. In a direct comparison, medial temporal ^11^C-UCB-J signal showed strongest correlations across all cognitive domains whereas for neocortical regions, ^18^F-FDG uptake seems more strongly correlated [39]. In mild cognitive impairment (MCI) there is a strong negative relationship between synaptic density and hippocampal tau accumulation [40, 41], while in MCI and AD, SV2A binding is also highly correlated with several cognitive domain functions [37, 40–42]. In FTD subtypes, which are mainly associated with aberrant TDP-43/tau/FUS protein depositions, distinct patterns of synaptic loss compared to other neurodegenerative disorders have been observed in populations including C9orf72 mutation carriers [43], behavioral variant FTD [44], PSP and CBD [45, 46]. Multimodal imaging has revealed an association between lower synaptic density and reduced functional connectivity, in addition to that accounted for by grey matter atrophy [47]. In Lewy body dementia, including Parkinson’s dementia and DLB, deposits of alpha-synuclein form the main underlying proteinopathy and compared to Alzheimer’s disease, there is often only mild atrophy. Also in this population, marked cortical synaptic loss can be observed [48, 49].

The short half-life of ^11^C (20.4 min) and single tracer manufacturing per subject makes the clinical routine use of ^11^C-UCB-J cumbersome. Recently, ^18^F-SynVesT-1, an optimized ^18^F-labeled analogue of UCB-J with similar kinetics, binding, and test-retest properties has been evaluated in humans [50–52] with also good quantitative correspondence to ^11^C-UCB-J.

The primary objective of this clinical trial is to determine the added value for clinical use of synaptic density imaging using a multimodal simultaneous PET/MR approach in patients with dementia and other patients developing cognitive dysfunction, by assessing the functional burden of the disease on the level of the synapse instead of synaptic activity/glucose metabolism, and by identifying an optimal combination of synaptic density and other PET/MR imaging metrics (perfusion, structural atrophy) that may allow early assessment and risk stratification for cognitive and behavioral dysfunction in de novo patients with uncertain origin of dementia. This will enable us to better understand the underlying pathophysiology of dementia, assess the direct consequences of underlying proteinopathy, relate this to subsequent structural measures and identify those parameters that can contribute to the accuracy of an early differential diagnosis. It will thereby aid the societal/economic challenge of earlier diagnosis, prognosis and biomarker development for more objective and more efficient monitoring of novel therapeutic trials. Secondary objectives are to assess how synaptic density is altered in the different cognitive disorders and how it correlates to specific symptomatology. Moreover, a direct comparison between ^11^C-UCB-J and ^18^F-SynVesT-1 in terms of distribution volume and standardized uptake value ratios (SUVR), including variability and noise levels will be conducted in HV.

## Methods and analysis

### Study design

This is a prospective and multimodal imaging study that aims to investigate and compare the discriminative power of ^18^F-SynVesT-1 PET and the standard-of-care ^18^F-FDG PET in differential diagnosis for cognitive disorders. Study approval has been obtained by the ethics committee at the University Hospitals Leuven and by the Belgian competent authority (federal agency for medicines and health products (FAMHP) as well as the federal agency for nuclear control (FANC)). It will be conducted in accordance with the latest version of the Declaration of Helsinki as well as the Good Clinical Practice guidelines and all applicable regulatory requirements. The collection, processing and disclosure of personal data, such as patient health and medical information is subject to compliance with applicable personal data protection and the processing of personal data (General Data Protection Regulation and the Belgian Law on the protection of natural persons with regard to the processing of personal data). The study has been registered on ClinicalTrials.gov (NCT05384353).

### Study hypotheses

The following specific research questions will be addressed:

(i) Can SV2A PET, using the novel tracer ^18^F-SynVesT-1, be approximated quantitatively by means of a simplified reference tissue model in HV and is it quantitatively comparable to ^11^C-UCB-J?
(ii) Are there regional differences between SV2A PET and ^18^F-FDG PET and do the patterns remain unchanged versus HV?
(iii) Can ^18^F-SynVesT-1 PET detect various forms of dementia as accurately as ^18^F-FDG PET?
(iv) Does a combination of ^18^F-SynVesT-1 PET with MR-based markers of structural abnormalities and arterial spin labeling allow a better discrimination of different forms of dementia?

Based on literature and current knowledge, the following hypotheses are formulated:

(i) Healthy aging does not result in reduced synaptic density in adults up to 85 years. ^18^F-SynVesT-1 may show a better signal-to-noise ratio and at least equivalent utility for clinical applicability compared to ^11^C-UCB-J.
(ii) Simplified quantification of ^18^F-SynVesT-1 PET is possible with white matter (centrum semiovale) as reference region [30, 53] or cerebellum as pseudo-reference region [42].
(iii) Synaptic density and arterial spin labeling, as indirect marker of neuronal activity, are related in absolute terms in healthy agers, while regional differences are present that may affect the sensitivity of detection of pathological consequences.
(iv) ^18^F-SynVesT-1 PET allows accurate detection of patients with an underlying pattern specific for Alzheimer’s disease, frontotemporal dementia and Lewy Body disease, and can discriminate these neurodegenerative diseases from healthy aging and patients with cognitive impairment due to late-life psychiatric disorder.
(v) ^18^F-SynVesT-1 PET has non-inferior sensitivity and specificity of diagnosis in comparison to ^18^F-FDG PET.
(vi) Non-invasive determination of absolute ^18^F-FDG glucose metabolism, ASL and SV2A (SUVR) allows discrimination of patients with late-life psychiatric disorder and neurocognitive disorders with highest accuracy for synaptic density imaging.
(vii) Synaptic density PET is better correlated to memory and executive neurocognitive tests compared to ^18^F-FDG PET or ASL, especially in the frontal and medial temporal cortex.

### Study participants

A total estimated cohort of 110 patients with uncertain diagnosis of cognitive impairment that are sent for ^18^F-FDG PET brain imaging in their clinical-diagnostic work-up will be recruited from the memory clinic of the University Hospitals Leuven, Belgium. This includes patients who do not yet have a final diagnosis at study inclusion but do show objectified cognitive impairment on neuropsychological evaluation as to avoid the inclusion of patients with subjective cognitive decline that have a lower likelihood of underlying neurodegenerative disease. All included patients will thus suffer from MCI (amnestic or non-amnestic) or dementia, according to the Diagnostic and Statistical Manual of Mental Disorders (DSM-V) criteria (respectively mild or major neurocognitive disorder) [54]. An initial working diagnosis is made by the clinician based on clinical workup, neuropsychological testing, biomarker tests (for AD suspicion: CSF Aβ42/Aβ40 and total tau or amyloid PET if no CSF) and sMRI. Patients with uncertain diagnosis are referred for ^18^F-FDG PET. From this population patients are recruited for the proposed research. ^18^F-SynVesT-1 scanning will be done as soon as possible, within maximally 3 months from ^18^F-FDG PET.

Patient inclusion criteria are:

(i) Patients referred with uncertain diagnosis of cognitive impairment and subsequent need for ^18^F-FDG PET in their work-up to differentiate between neurocognitive disorders or to exclude a neurodegenerative disorder in late-life psychiatric disorders with cognitive impairment.
(ii) A routine neuropsychological assessment has been performed during clinical work-up in the memory clinic.
(iii) Subject is at least 30 years old.

Main patient exclusion criteria are:

(i) Subject has a history or evidence of other major neurological, major psychiatric or major internal pathology (including cardiac, lung, hematological, gastro-intestinal disorders or most forms of cancer).
(ii) Subject’s neurological condition has a predominant impact on motor function.
(iii) Subject has no objective cognitive-behavioral deficit based on neuropsychological assessment.
(iv) Subject has important vascular changes abnormal for age or other structural lesions on MR.
(v) Subject is currently a user (including ‘’recreational use’’) of any illicit drugs, including cannabis, or has a history of drug or alcohol abuse.
(vi) Subject has a contra-indication for MRI scanning.
(vii) Subject suffers from claustrophobia or cannot tolerate confinement during PET-MRI scanning procedures; subject cannot lie still for 30 min inside the scanner.
(viii) Subject does not understand, is unwilling or unable to perform the study procedures or does not have a guardian who understands the study procedures.
(ix) Subject is pregnant (according to Ulti Med hCG urine test) or breastfeeding. For women of childbearing potential: subject does not not agree to apply appropriate contraception methods during study participation and continues to do so for at least 6 months after study completion.

For comparison, we will also include a sample of 40 HV between 18 and 85 years old who have no prior or current medical, psychiatric or neurologic illness as determined by review of medical history as well as extended clinical and neuropsychological assessment. In addition, for subjects below 60 years of age, an unremarkable structural MRI scan is expected whereas for subject above 60 years of age, a Fazekas of < 2 is accepted. Main exclusion criteria of patients also apply to the HV series. However, subjects on anticoagulant therapy or who chronically use medication with effects on the central nervous system will be excluded from the HV sample. Of this HV cohort, 10 subjects will also be subjected to a ^11^C-UCB-J PET in order to assess the quantitative comparability with ^18^F-SynVesT-1.

#### Sample size

Taking into account the test-retest variability for ^18^F-SynVesT-1 (5–10%) [52]; and a non-inferiority margin of 10%, assuming an α-level of 0.025 and a statistical power of 80%, the required sample size is 8 patients, of no ‘true’ difference. As sensitivity analysis, in case this difference would be 1% or 2%, sample sizes would increase to 10 and 12 per group.

Moreover, the sample size for brain receptor single-center PET studies using absolute quantification as we do in healthy subjects is typically between 10 and 20 in most literature studies. We include 40 healthy controls and 110 patients with estimated subgroups sizes of 50 amnestic mild cognitive impairment (aMCI)-AD, 20 FTD, 20 DLB and 20 patients with late-life psychiatric disorder based on our referral data.

#### Recruitment

A total of 110 patients (M/F) will be recruited with uncertain diagnosis of cognitive impairment, as consecutively sent for ^18^F-FDG PET-MR imaging in their workup (estimated subgroup size: 50 patients with aMCI-AD; 20 patients with DLB; 20 patients with FTD; 20 patients with late-life psychiatric disorder). A minimal age was set at 30 years old, but the expected age ranges from 50 until +/- 85y old. The neuropsychological testing is expected to already be obtained during the diagnostic assessment. Some additional tests on top of those from the memory/referral clinic may be foreseen if necessary to ensure coverage of all relevant domains. The main sources of referrals constitute the UZ Leuven memory clinic of neurology, psychiatry, a collaboration with geriatrics and external hospitals situated in proximity of Leuven.

For the recruitment of HV, we will make use of (I) advertisements in targeted local journals and on the bulletin boards in the buildings of KU Leuven and UZ Leuven, on the websites of UZ/KU Leuven and in social media, and (II) of the volunteer database of the Center for Clinical Pharmacology. Forty screened HV (M/F) aged 18–85, approximately evenly spread over decades, will be included. These volunteers are screened for general good health condition (based on medical history, physical examination including vital parameters and clinical laboratory test and urinalysis), for neurocognitive and executive functioning via neuropsychological assessment, for amyloid status with amyloid PET scan (^18^F-NAV4694) if > 50y (below amyloid negativity is presumed) and for existence of other possible exclusion criteria.

#### Screening

Study participants will first be subjected to a screening visit during which the study protocol will be explained in detail. Thereafter, the informed consent form will be signed by all participants– or their legal representative in case of patients who are unable to give consent themselves– prior to study inclusion. In HV, a Mini-Mental State Examination (MMSE) will be used to assess global cognition, and depressive symptoms will be screened through the Beck Depression Inventory (BDI). Furthermore, to assure general good health in this group a thorough blood analysis and urinalysis are executed with assessment of hematology, coagulation and chemistry for blood and pH, hematuria, leukocyturia, glucosuria, proteinuria and epithelial cells for urine.

### Data collection and analysis

#### Imaging

All scans will be performed at the Nuclear Medicine PET-MR unit of the University Hospital Leuven, on a General Electric (GE, Milwaukee, WI, USA) Signa PET-MR (time-of-flight (TOF) PET, 3-Tesla MR) scanner. A validated zero echo time (ZTE) sequence will be used for attenuation correction [55]. ZTE allows MR imaging of bone, which enables better skull segmentation, tissue assignment and accurate PET quantification. All tracers will be produced in-house.

##### ^18^F-SynVesT-1 PET-MR

During the first scan visit, the ^18^F-SynVesT-1 PET-MR scan will be acquired as a measure of synaptic density (SV2A). The tracer will be administered through a venous catheter with an intended dose of 150 MBq (± 10%).

For patients, a static 30-minute PET acquisition will be conducted, starting 60 min post injection. For HV, an arterial catheter line will be placed in a radial artery for arterial blood sampling. The scanning protocol consists of a dynamic PET acquisition of 90 min starting at time of injection, with arterial input function via arterial blood samples for full kinetic modelling and investigation of the reference region. Discrete blood samples will be manually collected every 10s from 10 to 90s; every 15s from 90s to 3 min; and then at 3.5, 5, 6.5, 8, 12, 15, 20, 25, 30, 45, 60, 75 and 90 min. Plasma samples for correction of radioactive metabolites will be collected at 3, 8, 15, 30, 60 and 90 min after injection.

For all participants, data will be obtained in list mode and will be reconstructed using an ordered subset expectation maximization (OSEM) algorithm (4 iterations and 28 subsets), with corrections for scatter, random coincidences, deadtime, radioactive decay, and ZTE-based attenuation as described above and with post-filtering with a 3D isotropic gaussian of 4 mm Full Width at Half Maximum (FWHM). ^18^F-SynVesT-1 PET acquisitions for HV will be rebinned into 30 frames (6 × 15 s; 3 × 30 s; 3 × 1 min; 3 × 3 min; 15 × 5 min). ^18^F-SynVesT-1 PET acquisitions for patients will be rebinned into 6 frames of 5 min.

Simultaneously with PET, following MR measures will be acquired from all participants using a vendor-supplied Nova 32 channel coil: structural 3D T1-weighted MR, 3D T2-weighted fluid-attenuated inversion recovery (FLAIR) MR, resting state functional-MRI and triple-plane tagged enhanced ASL perfusion.

##### ^11^C-UCB-J PET-MR

To assess quantitative similarity of ^18^F-SynVesT-1 and ^11^C-UCB-J, both targeting SV2A, 10 HV will additionally receive a dynamic ^11^C-UCB-J PET acquisition 0–90 min post injection using PET-MR, including arterial sampling, following the same acquisition and reconstruction protocol as for ^18^F-SynVesT-1. Again, the tracer will be administered through a venous catheter with an intended dose of 300 MBq (± 10%). No additional MR sequences will be acquired.

##### ^18^F-NAV-4694 PET-MR

^18^F-NAV-4694 PET (formerly known as ^18^F-AZD-4694) has been shown to selectively bind amyloid-β plaques in AD [56] and can accurately determine cerebral amyloid status [57]. Its comparability to the most widely used ^11^C-PiB PET tracer has been demonstrated [58]. For all included HV above 50 years, known cerebral amyloid status is required for inclusion in the study (max 1 year old). If amyloid status is unknown or was not determined recently, a routine 20-minute amyloid PET scan (^18^F-NAV-4694) will be acquired, starting 40 min post injection. This tracer will also be administered through a venous catheter with an intended dose of 120 MBq (± 10%). Data will be obtained in list mode and will be reconstructed using an ordered subset expectation maximization (OSEM) algorithm (2 iterations and 32 subsets), with corrections for scatter, random coincidences, deadtime, radioactive decay, and ZTE-based attenuation as described above and with post-filtering with a 3D isotropic gaussian of 4.5 mm FWHM. No additional MR sequences will be acquired. For practical reasons, ^18^F-NAV-4694 could also be acquired on a PET-CT scanner (TruePoint, Siemens, Erlangen, Germany) and reconstructed using ordered subsets expectation maximization (3 iterations and 21 subsets), with corrections for scatter, random coincidences, deadtime, radioactive decay, and CT-based attenuation, with post-filtering with a 3D isotropic gaussian of 2 mm FWHM.

Amyloid status will be determined by visual interpretation and classified as positive or negative by an expert in nuclear medicine (K.V.L.).

#### Neuropsychological assessment

For HV, the neuropsychological testing for this study consists of a cognitive test battery with subtests of the Cambridge Neuropsychological Test Automated Battery (CANTAB) [59]: motor screening task (MST), reaction time (RT), paired association learning (PAL) and spatial working memory (SWM). To obtain a more complete evaluation of all cognitive domains this also includes the Mini-mental state examination (MMSE) [60] or Montreal Cognitive Assessment (MoCA) [61], Clinical Dementia Rating Scale (CDR) [62], Rey auditory verbal learning test (RAVLT) [63], Boston naming task (BNT) [64], trail making test A and B (TMT) [65], Raven’s colored progressive matrices (RCPM) [66] and animal verbal fluency (AVF) [67]. To screen for depressive, anxious and other important psychiatric symptoms we will use the Symptom Checklist-90 (SCL-90) [68], Beck depression inventory (BDI) [69] and Geriatric depression scale (GDS) [70].

For patients, in case some of the above test domains have not been established, the study team will determine which additional tests of the above on top of the routine neuropsychological testing should be included, if relevant for the suspected disease and also on the base of clinical examination and ^18^F-FDG PET.

#### Data analysis and statistics

All reconstructed ^18^F-SynVesT-1 and ^11^C-UCB-J PET data will be corrected for motion artefacts using PMOD software (v4.1, PMOD Inc. Zurich, Switzerland). The frames of static acquisitions will be averaged and rigidly co-registered to the corresponding T1-weighted MRI. For partial volume correction, a region-based voxel-wise (RBV) correction, which is based on the Yang approach [71–73] will be applied as implemented in an in-house validated pipeline in Python 3.9 [74]. Data analysis will be done using volume-of-interest (VOI) analysis using FreeSurfer 6.0 or python 3.9 for anatomical parcellation and delineation of the VOIs. For voxel-based analysis, PET data will be spatially normalized to template space using a non-linear normalization as obtained by the CAT12 toolbox of statistical parametric mapping (SPM12; Welcome Trust Centre for Neuroimaging, University College London, UK) and smoothed using an isotropic Gaussian kernel with 8 mm FWHM (voxel size: 1.5 × 1.5 × 1.5 mm).

^18^F-SynVesT-1 and ^11^C-UCB-J uptake in HV will be quantified by distribution volume (V_T_) using a one-tissue compartment model as previously validated [26, 52]. For the tracer kinetic modeling of ^18^F-SynVesT-1 in HV, the centrum semiovale will be investigated as a reference region [30, 53] as well as the cerebellum as a pseudo-reference region [42]. For ^18^F-SynVesT-1 patient data, standardized uptake value ratios (SUVR) will be calculated after this reference region exploration in HV.

The evaluation will be done in 3 steps: firstly, referring physicians will be asked to provide suspected diagnoses and confidence scores at predefined moments during clinical workup. Secondly, anonymized scans (orthogonal images, 3D surface rendering and z-score images by comparison to in-house database as clinical routine / comparison to healthy control normal data) with core clinical information will be presented at regular intervals to 3 nuclear medicine physicians with variable experience but after a short reader training in healthy ^18^F-FDG and ^18^F-SynVesT-1 scans. They will provide an image-based diagnosis and indicate the corresponding level of certainty. The different visual readers will then provide a consensus protocol for each scan. After visual reading, also semiquantitative volume of interest- and voxel-based classifications will be carried out (for ^18^F-FDG, ^18^F-SynVesT-1, ASL and rMAPS). Lastly, data will be compared in terms of accuracy based on the consensus clinical diagnosis (about 6 months after ^18^F-FDG scanning). Consensus clinical diagnosis is based on initial clinical findings and evolution, CSF biomarkers (/ amyloid PET), sMRI and ^18^F-FDG PET results. Note that patient data analysis can only be initiated after the finalization of the healthy subject ^18^F-SynVesT-1 database (so z-score images can be obtained).Voxel-based morphology (VBM) and voxel-based cortical thickness (VBCT) analysis, adjusted for total intracranial volume will be used to assess structural gray matter volume and thickness differences in all subjects in the CAT12 toolbox of Statistical Parametric Mapping (SPM12). We will analyze individual imaging modalities (^18^F-SynVesT-1 and ^18^F-FDG PET, ASL, structural MR, rs-fMRI) independently in a univariate analysis to address the different modality, as well as using a multivariate approach as part of the underlying research questions.

Statistical analyses will be performed using GraphPad Prism version 9 (GraphPad Software, La Jolla, CA). Demographic characteristics will be compared between groups using an unpaired Student t-test, unpaired Mann-Whitney U test, Fischer’s exact test or Chi-square test for trend as appropriate. Data normality will be verified by a Shapiro-Wilk test (α = 0.05). Correlation analyses will be performed by a Pearson correlation if data are normally distributed or by a Spearman correlation if data do not follow a normal distribution. Paired student’s t-tests or a non-parametric substitute will be performed in both a voxel-based and a VOI-based analysis to investigate regional differences between ^18^F-SynVesT-1 PET and ASL. Signature patterns of reduced synaptic density for different cognitive disorders will be assessed by comparing ^18^F-SynVesT-1 in patient subgroups versus HV using Student’s t-tests or Mann Whitney U-tests. To explore the diagnostic performance of ^18^F-SynVesT-1 compared to ^18^F-FDG, scans will be visually assessed by different specialists in nuclear medicine/neurologists and diagnostic accuracy (sensitivity, specificity and accuracy) as well as the interobserver agreement will be calculated. All results will be adjusted for multiple comparisons (Bonferroni) and investigated two-sided at a significance level of 0.05.

## Discussion

This study will compare the diagnostic accuracy of ^18^F-SynVesT-1 to ^18^F-FDG PET in cognitive disorders with uncertain etiology and in exclusion of a neurodegenerative cause in patients with cognitive impairment in late-life psychiatric disorders. The acquisition of PET and MR imaging data as well as neuropsychological testing both in patients and HV will enable us to assess not only the relationship between cognition and imaging data but also between these different imaging modalities (PET and ASL) themselves.

Using ^11^C-UCB-J, synaptic density has been assessed in AD, FTD and DLB, compared to HV. For AD, largest effect sizes are found in the hippocampus and other medial temporal regions extending into the posterior cingulate cortex and to a lesser extent in neocortical regions [36], whereas in this population, ^18^F-FDG showed larger effect sizes especially in neocortical regions [38]. In patients with DLB, synaptic loss was observed in substantia nigra, occipital, parietal and frontal cortices but not in medial temporal regions such as hippocampus and amygdala [48]. In a direct comparison of ^11^C-UCB-J and ^18^F-FDG in DLB patients, the magnitude as well as spatial extent of hypometabolism exceeded that of synaptic loss [75]. As for FTD, decreased synaptic density was most prominent in frontal regions and to a somewhat lesser extent also in temporal regions, insula and anterior cingulate [44]. To the best of our knowledge, no direct comparison with FDG PET has been published in FTD. Although pilot studies discriminating AD from HV show larger effect sizes for hypometabolism compared to synaptic loss, regional changes e.g. in the hippocampus, may show increased specificity and effect size itself is not prohibitive to an equivalent or even better discrimination between the different dementia subtypes.

Since patients will be included upon referral for ^18^F-FDG PET at the UZ Leuven PET center, the sample will be highly representative for a real-life clinical setting. Another strength of our study is the use of simultaneous PET and MR imaging, which will allow us to investigate multiple modalities with limited associated burden for patients. We believe that achieving the anticipated sample size in an adequate time window is feasible due to this 1-scan study protocol in combination with the considerable extent of the local hospital memory clinic. ^18^F-SynVesT-1 PET will not only provide a cleaner marker of synaptic density, but it also obviates the need (i) for patients to be fasted at least four hours prior to tracer injection, (ii) to delay simultaneous MR scanning to avoid primary auditory cortex activation and (iii) to provide a dark environment prior to scanning to limit visual cortex activation. Furthermore, as visual stimulation has been shown not to change ^11^C-UCB-J levels in the occipital cortex [76], no influence of scanning with eyes open or closed is expected.

As for limitations and compromises made in the design of the study, some sources of bias may be present. Expert visual readers will be more experienced in reading ^18^F-FDG scans compared to ^18^F-SynVesT-1 scans. Therefore, they will be presented a normal dataset for visual inspection before patient reads. Due to clinical need and the importance of ^18^F-FDG PET in the diagnostic work-up, the ^18^F-FDG PET result will likely influence the final clinical diagnosis and might result in a bias in favor of ^18^F-FDG PET. Therefore, anonymized reads will be done also by expert nuclear medicine physicians not involved in the clinical workup.

The sample size calculation was based on observed effect sizes for ^11^C-UCB-J as ^18^F-SynVesT-1 data are not yet publicly available. However, similarity of ^11^C-UCB-J and ^18^F-SynVesT-1 in terms of distribution volume and binding potential as well as test-retest characteristics has been demonstrated [51, 52]. Accordingly, it can be expected to find similar effect sizes for ^18^F-SynVesT-1 as for ^11^C-UCB-J and we believe the anticipated sample size will result in adequate power. Another limitation will be the short acquisition protocol for the patient group and the associated simplified quantification method (SUVR), which was a trade-off between scanning a larger sample with simplified quantification or a smaller sample with full dynamic quantification. Of note, performing 90-minute dynamic acquisitions in a clinical setting is not feasible. As we aim to investigate the clinical applicability of ^18^F-SynVesT-1, the use of short static acquisitions is justified. The single-tracer nature of the study can be seen as a limitation since we cannot for example determine correlations with tau or amyloid PET imaging data. Nevertheless, ^18^F-FDG PET data will be acquired for clinical work-up in all patients (as inclusion criteria) and most patients will also receive lumbar punctions to determine cerebrospinal fluid levels of Aβ42/Aβ40 and total tau to exclude or confirm AD. Therefore, performing retrospective (sub)analyses using these data might also be possible upon study completion and ethics approval.

In conclusion, this study will provide further insight into synaptic density PET and its diagnostic applicability in a clinical routine setting.

## Data Availability

Anonymized data will be deposited in an access-controlled file server used by the academic research PET imaging group, which can be shared upon reasonable request from any qualified investigator and on approval by the local Ethics Committee.

## Declarations

### Study status

The study is currently ongoing, and recruitment of study participants has started. Data collection has commenced and is estimated to be completed late 2024.
